# Parliament and Publications

**Published:** 1910-10-08

**Authors:** 


### PARLIAMENT AND PUBLICATIONS.

### DEATH-RATE AMONG SEAMEN.

The total number of seamen (belonging to merchant vessels registered in the United Kingdom) whose deaths were reported during the year ended June 30, 1909, was 2,143, or 1 in 111. The number of deaths by injury was 1,157, or 1 in 206, and the number of deaths from disease 986, or 1 in 241. The death-rate from injury among the crews of sailing-vessels (1 in 74) was much greater than among the crews of steamers (1 in 253); but the death-rates on board sailing-ships and steamers were less dissimilar in respect of loss of life by disease than by injury, the loss from disease in sailing-vessels being 1 in 236, and in steamers 1 in 242. The death-rate from suicide among stokehold hands was about five times as high as among engine-room hands, and more than three times as high as among persons outside the engineers' department. Drink was reported to be the direct cause of the deaths of twelve seamen, of whom eight were British and four were foreigners. From the reports received it is probable, however, that drink was a contributory cause of the deaths of 126 persons in all. The causes of deaths from diseases were as follows: Specific febrile diseases, 241; constitutional diseases, 135; diseases of the nervous system, 85; diseases of the circulatory system, 137; diseases of the respiratory system, 139; diseases of the digestive system, 123; diseases of the urinary system, 46; debility and ill-defined and unknown diseases, 70.—*Deaths of Seamen*, Cd. 5377. Price 9d.

